# Histologic Activity Modifies the Predictive Value of Systemic Inflammatory Markers in Ulcerative Colitis

**DOI:** 10.3390/diagnostics16142191

**Published:** 2026-07-14

**Authors:** Minjee Kim, Ji Eun Kim, Eun Ran Kim, Sung Noh Hong, Dong Kyung Chang, Young-Ho Kim

**Affiliations:** Department of Medicine, Samsung Medical Center, School of Medicine, Sungkyunkwan University, Seoul 06351, Republic of Korea; minjee0803.kim@samsung.com (M.K.);

**Keywords:** ulcerative colitis, neutrophil-to-lymphocyte ratio, histologic activity, relapse, biomarkers

## Abstract

**Background/Objectives:** Systemic inflammatory markers may predict relapse in ulcerative colitis (UC), but whether histologic activity modifies their prognostic value remains unclear. We evaluated whether baseline neutrophil-to-lymphocyte ratio (NLR), lymphocyte-to-monocyte ratio (LMR), and neutrophil-to-platelet ratio (NPR) predict clinical relapse in UC patients with endoscopic improvement and whether these associations differ by histologic status. **Methods:** This retrospective cohort study included UC patients with Mayo Endoscopic Subscore 0 or 1. Histologic status was assessed using the Geboes score and classified as histologic improvement (<3.1) or histologic activity (≥3.1). Baseline NLR, LMR, and NPR were calculated. Clinical relapse was the primary outcome. Cox proportional hazards models and Kaplan–Meier analyses were performed. **Results:** Histologic activity was present in 132 patients (30.3%). Baseline inflammatory indices did not differ between groups. Higher baseline NLR was associated with relapse in the histologic activity subgroup (hazard ratio [HR], 1.72; 95% confidence interval [CI], 1.06–2.92; *p* = 0.028), but not in the histologic improvement subgroup (HR, 0.98; 95% CI, 0.70–1.38; *p* = 0.901). After adjustment for endoscopic activity, high NLR remained independently associated with relapse in patients with histologic activity (HR, 1.83; 95% CI, 1.10–2.05; *p* = 0.021), with significant interaction by histologic status (*p* = 0.019). LMR and NPR were not associated with relapse. **Conclusions:** Histologic activity modifies the prognostic value of baseline NLR in UC patients with endoscopic improvement. Combining NLR with histologic assessment may improve relapse risk stratification.

## 1. Introduction

Ulcerative colitis (UC) is a type of chronic inflammatory bowel disease. Although the number of treatment options has increased and treat-to-target approaches have become more precise, the relapsing-remitting disease course itself necessitates escalation of medication, hospitalization, and potentially surgical interventions. Therefore, it is crucial to effectively manage ulcerative colitis as a chronic disease to prevent relapse. Management is highly complex and involves monitoring numerous indicators. These include patient symptoms, endoscopic findings, inflammatory marker levels, and histological parameters.

The Mayo Endoscopic Sub-score (MES) was used as an endoscopic indicator, and an endoscopic improvement score of 0 or 1 indicated mucosal healing. However, distinguishing between MES 0 and 1 is challenging, and traditional endoscopic mucosal healing does not indicate a complete absence of inflammation [1]. Achieving deeper histological remission has become the treatment goal. Concurrently, interest has grown in noninvasive tests, particularly systemic inflammatory markers detected in blood tests, as they are simple and affordable. Conventional inflammatory markers such as C-reactive protein (CRP) and erythrocyte sedimentation rate (ESR) are used to monitor the disease but have low specificity and sensitivity in UC [2]. In addition, fecal calprotectin is expensive, and patients may find it inconvenient to bring stool samples to the hospital when undergoing the test.

The neutrophil-to-lymphocyte ratio (NLR) has emerged as a potential biomarker. Several reports have suggested that an elevated baseline NLR may predict unfavorable outcomes in patients with cancer, disease activity in rheumatoid arthritis, and prognosis in patients with sepsis [3,4,5,6]. Moreover, previous studies have demonstrated that NLR correlates with disease activity in UC, and mean NLR values are elevated during active inflammation [7,8,9]. Moreover, NLR is an independent prognostic factor for infliximab and tacrolimus therapy [10,11]. Previous studies have also reported that in patients with an MES of 0 or 1, a higher NLR is associated with a higher risk of clinical relapse [7].

More recently, additional blood count–derived indices such as the lymphocyte-to-monocyte ratio (LMR) and neutrophil-to-platelet ratio (NPR) have been proposed as alternative markers of systemic inflammation. Monocytes can differentiate into macrophages and dendritic cells within the tissues. During inflammatory conditions, pro-inflammatory cytokines and chemokines stimulate monocyte production in the bone marrow and promote their recruitment to sites of inflammation, where they further differentiate into tissue-resident macrophages and dendritic cells. Persistent monocyte activation and impaired innate immune responses have been implicated in the pathogenesis of inflammatory bowel disease (IBD), which may explain the presence of absolute monocytosis in patients with active UC [12]. Accordingly, monocyte counts are expected to increase during active inflammation [13]. Cherfane et al. observed that an elevated absolute monocyte count and low lymphocyte-to-monocyte ratio (LMR) can predict disease activity in patients with UC [14].

Neutrophils and platelets are present in the histology of patients with active UC [15,16]. Platelets contribute to the inflammatory processes by storing interleukin-8 (IL-8), a key neutrophil chemoattractant, and expressing p-selectin [17]. This leads to the overproduction of superoxide and the formation of neutrophil-platelet aggregates, which are elevated in patients with UC. Furthermore, in dextran sodium sulfate-induced colitis models, inhibition of these aggregates has been shown to suppress colonic inflammation [18,19]. Previous research has indicated that the neutrophil-to-platelet ratio (NPR) exhibits a strong correlation with disease activity, along with superior sensitivity and specificity. Furthermore, its significant association with the anatomical extent of the disease makes NPR a more efficacious and reliable biomarker for the clinical assessment of UC [20].

Persistent microscopic inflammation may represent a biological context in which systemic inflammatory indices reflect ongoing disease processes more accurately, whereas their predictive value may be attenuated in patients in histological remission. However, few studies have examined whether histological activity modifies the association between baseline inflammatory markers and clinical relapse in UC.

Therefore, this study aimed to evaluate whether baseline blood count-derived inflammatory indices, including NLR, LMR, and NPR, predict clinical relapse in patients with UC who have achieved endoscopic improvement, defined as MES 0 or 1. We further aimed to determine whether the prognostic value of these markers differs according to baseline histologic status, thereby clarifying whether histologic activity modifies the association between systemic inflammatory markers and subsequent relapse.

## 2. Materials and Methods

### 2.1. Study Design, Setting, and Participants

This was a retrospective cohort study conducted at Samsung Medical Center, a tertiary institution in Seoul, South Korea. In this center, tissue sampling from the most inflamed areas and the rectum has been routinely performed since 2018, even with inactive endoscopic activity. The study enrolled patients with an MES 0 or 1 between January 2018 and December 2019. The exclusion criteria were absence of histological examination at the index colonoscopy, inadequate histological assessment, age <18 years, prior colorectal cancer, prior colectomy, and insufficient follow-up. Histological assessment was considered inadequate when no biopsy specimen was available, when the specimen was insufficient for histological grading, when the pathology slide or report was unavailable, or when the Geboes score could not be reliably assigned because of poor specimen quality or non-evaluable tissue. The Geboes score used for this study was assigned retrospectively for research purposes by an experienced gastrointestinal pathologist blinded to clinical outcomes and laboratory data. Only patients who underwent histological evaluation at the index colonoscopy and had adequate biopsy specimens available for Geboes scoring were eligible for inclusion. The histological activity was assessed using the Geboes grading system. Patients were stratified according to histologic activity status into histologic improvement (HI+, Geboes score < 3.1) and histologic activity (HI−, Geboes score ≥ 3.1). The patients were divided into two groups according to histologic activity at the index colonoscopy. Patients who underwent the first follow-up colonoscopy and had complete laboratory data were included in the first colonoscopy follow-up analysis. Complete blood tests with differential white blood cell (WBC) counts were performed by electronic cell counting.

Electronic medical records were reviewed to obtain inclusion, exclusion, and follow-up variables for each patient. The study was approved by the Institutional Review Board of Samsung Medical Center (IRB No. SMC 2026-02-026-002). The IRB of Samsung Medical Center granted an exemption from informed consent because all data were analyzed anonymously. The study protocol conformed to the ethical guidelines of the 1975 Declaration of Helsinki as reflected in a priori approval by the institution’s human research committee.

### 2.2. Baseline Variables and Definitions

Baseline demographic and clinical variables included age, sex, disease duration, and endoscopic disease activity assessed using the Mayo Endoscopic Subscore (MES) and C-reactive protein (CRP) level. Baseline inflammatory indices, NLR, LMR, and NPR, were calculated using laboratory values obtained at the time of the index assessment. MES, a component of the Mayo score, classifies mucosal inflammation based on a 4-point scale from 0 to 3 according to endoscopic findings (0: normal; 1: erythema, decreased vascular pattern, and mild friability; 2: marked erythema, absent vascular pattern, friability, and erosions; and 3: ulceration and spontaneous bleeding). Endoscopy was performed by various endoscopic specialists at this center; however, MES was reevaluated separately by three different specialists. The specialists were blinded to clinical data during the study period. Biopsy specimens were obtained as part of routine clinical practice. Biopsy specimens were obtained as part of routine clinical practice. At the index colonoscopy, biopsies were routinely obtained from the most inflamed area and from the rectum. In patients without definite endoscopic inflammation, biopsies were obtained from representative colonic mucosa and the rectum. This institutional biopsy approach has been routinely applied since 2018, including in patients with inactive or mildly active endoscopic findings. For the present study, histological activity was assessed retrospectively using the Geboes grading system by an experienced gastrointestinal pathologist who was blinded to clinical outcomes and laboratory data. Pathological findings were reviewed by an experienced pathology specialist and recorded using the Geboes score. The Geboes score classifies structural (architectural) changes as grade 0, chronic inflammatory infiltrate as grade 1, lamina propria neutrophils and eosinophils as grade 2, neutrophils in the epithelium as grade 3, crypt destruction as grade 4, and erosion as ulceration as grade 5. All endoscopic and pathological reviewers were blinded to the clinical information. Endoscopic remission was defined as an MES of 0, and histological improvement (HI) was defined as a Geboes score < 3.1. Medication use at the index colonoscopy was reviewed from the electronic medical records. Conventional immunomodulators included thiopurines, such as azathioprine and 6-mercaptopurine, and methotrexate where applicable. Clinical relapse was defined as a change or escalation of medication, hospitalization, or total colectomy owing to the aggravation of UC [21]. Clinical remission (CR) at the time of the index colonoscopy was defined as a patient-reported outcome (PRO), composed of a rectal bleeding sub-score of 0 (no rectal bleeding) and a stool frequency sub-score of 0 (normal stool frequency) or 1 (1 or 2 more daily stools than normal). Each leukocyte type is expressed as a percentage of the total number of WBCs. NLR was calculated from the differential count by dividing the absolute neutrophil count by the absolute lymphocyte count. LMR was calculated from the differential count by dividing the absolute lymphocyte count by the absolute monocyte count. NPR was calculated by dividing the absolute neutrophil count by the platelet count.

### 2.3. Outcome Definition

The primary outcome was clinical relapse, which was defined as relapse during follow-up. Relapse-free survival time was calculated from the index date to the date of clinical relapse or last follow-up.

### 2.4. Statistical Analysis

Baseline characteristics were summarized for the entire cohort and according to histologic activity status (HI+ vs. HI−). Continuous variables are presented as medians with interquartile ranges (IQR) and compared using the Wilcoxon rank-sum test. Categorical variables were expressed as numbers (percentages) and compared using the chi-square test or Fisher’s exact test, as appropriate.

NLR, LMR, and NPR were initially analysed as continuous variables in the univariable Cox regression analyses. For Kaplan–Meier analyses and clinically interpretable subgroup comparisons, these markers were dichotomised into high and low groups using the overall cohort median values, because universally validated cut-off values for predicting relapse in patients with UC and endoscopic improvement have not been established.

Variables included in the Cox regression analyses were selected a priori based on clinical relevance, previous literature, and availability in the baseline dataset. These variables included the inflammatory indices of interest (NLR, LMR, and NPR) and clinically relevant covariates, including age, sex, age at diagnosis, disease duration, MES, and CRP level.

Hazard ratios (HRs) and 95% confidence intervals (CIs) were calculated. Kaplan–Meier curves were generated, and log-rank tests were performed to compare relapse-free survival according to the inflammatory marker status stratified by histologic activity.

All statistical analyses were performed using R software 2016.01.0 version, and a two-sided *p*-value < 0.05 was considered statistically significant.

## 3. Results

### 3.1. Baseline Characteristics

**Figure 1:**
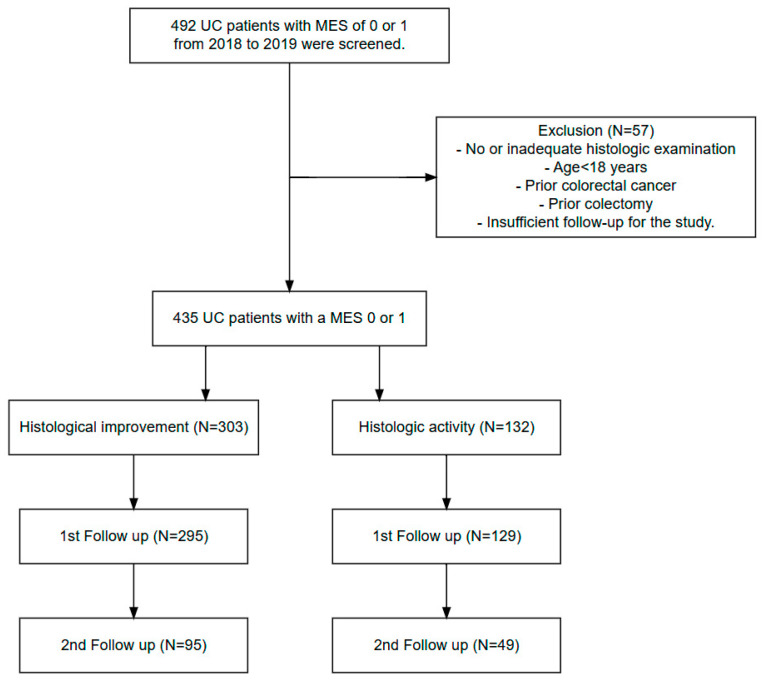
Study flow diagram. Patients with ulcerative colitis and Mayo Endoscopic Subscore 0 or 1 were screened between 2018 and 2019. After exclusions, 435 patients were included and stratified according to histologic improvement (Geboes score < 3.1) or histologic activity (Geboes score ≥ 3.1). Follow-up cohorts are shown.

A total of 492 patients with an MES of 0 or 1 were screened between January 2018 and December 2019 (Figure 1). The patients were divided into two groups according to histologic activity at the index colonoscopy. There were 303 and 132 patients in the histologically improved and active groups, respectively. At the second follow-up colonoscopy, 95 patients in the histologic improvement group and 49 patients in the histologically active group underwent complete blood tests.

The baseline characteristics of the study population are summarized in Table 1. The median age of the overall cohort was 48.0 years (IQR, 37.0–58.0), and 52.4% were male. The median age of patients with histologic activity (Geboes score > 3.1) was 43.0 years (IQR 33.0–56.0) and the duration was 29.45 months (IQR, 23.25–39.90). The median age of patients with histologic improvement (Geboes score < 3.1) was 50 years (IQR, 38–60) and the median disease duration was 37.20 months (IQR, 26.45–45.75). The proportions of patients with MES = 1 were 41.9% and 89.4% in the histologically improved and histologically active groups, respectively.

Baseline inflammatory markers, including the NLR, LMR, and NPR, did not differ significantly between the two histological subgroups. Median baseline NLR was 1.72 (IQR, 1.23–2.22), median LMR was 4.89 (IQR, 3.71–6.15), and median NPR was 0.01 (IQR, 0.01–0.02). The follow-up laboratory values for NLR, LMR, and NPR were 1.52, 4.85, and 0.01 respectively. Second follow-up laboratory values were 1.58, 4.89, and 0.01, respectively.

### 3.2. Univariable Analysis According to Histologic Activity

The results of the univariate Cox regression analyses stratified by histological activity are shown in Table 2.

In the histologically active group, a higher baseline NLR was significantly associated with an increased risk of clinical relapse (HR, 1.720; 95% CI, 1.062–2.921; *p* = 0.028). In contrast, NLR was not associated with relapse risk in the histological improvement group (HR, 0.98; 95% CI, 0.697–1.375; *p* = 0.901).

Baseline LMR and NPR were not significantly associated with clinical relapse in either histological subgroup. Age, sex, disease duration, and CRP levels also showed no significant association with relapse risk. MES was significantly associated with relapse only in the histological improvement subgroup (HR, 1.61; 95% CI, 1.141–2.275; *p* = 0.007) and was therefore included as a covariate in the multivariable analysis.

### 3.3. Multivariable Analysis Adjusting for Endoscopic Activity

To investigate the interaction effect between histological activity and inflammatory markers, multivariate Cox regression analyses adjusted for MES were performed (Table 3). Median baseline NLR was 1.72, median LMR was 4.89, and median NPR was 0.01. Values greater than the median were classified as high, whereas values equal to or lower than the median were classified as low.

In the histologically active subgroup, a high baseline NLR remained independently associated with an increased risk of clinical relapse after adjusting for MES (HR, 1.83; 95% CI, 1.095–2.045; *p* = 0.021). In contrast, the NLR was not associated with relapse risk in the histological improvement subgroup (HR, 0.958; 95% CI, 0.682–1.347; *p* = 0.805), and the interaction effect between histological activity and inflammatory markers was significant (*p* = 0.019).

The baseline LMR and NPR were not independently associated with clinical relapse in either subgroup after adjustment. No significant interaction effects were observed between the histologic activity status and LMR or NPR.

### 3.4. Clinical Relapse-Free Survival Analyses

**Figure 2:**
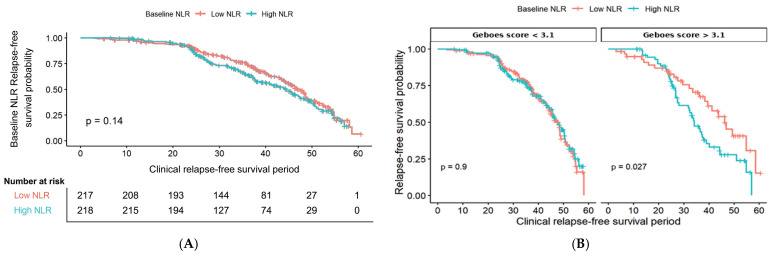
(**A**) Relapse-free survival according to median baseline neutrophil-to-lymphocyte ratio in the overall cohort. The *p* value was calculated using the log-rank test. (**B**) Relapse-free survival according to median baseline neutrophil-to-lymphocyte ratio stratified by histologic status. Relapse-free survival was shorter in patients with high baseline NLR in the histologic activity group but not in the histologic improvement group. *p* values were calculated using the log-rank test.

**Figure 3:**
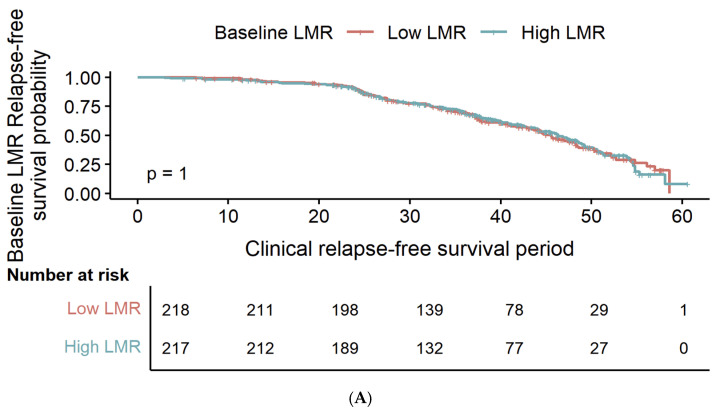

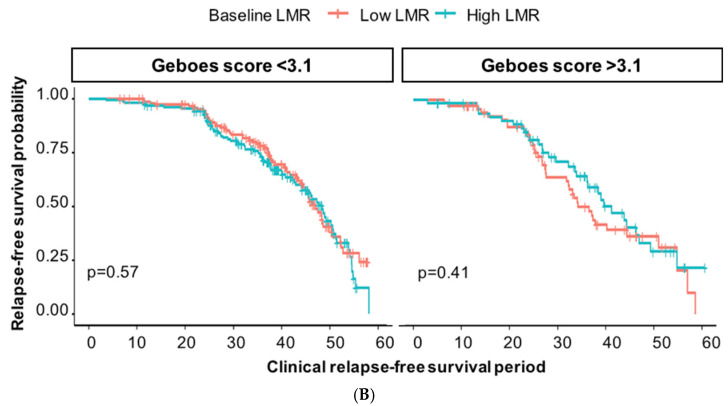

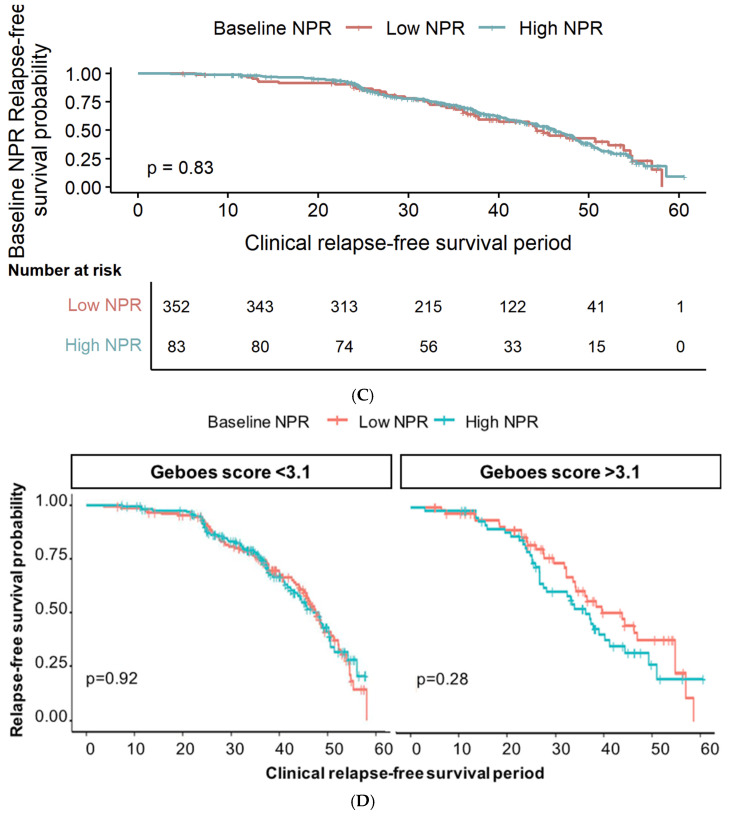
(**A**) Relapse-free survival according to median baseline lymphocyte-to-monocyte ratio in the overall cohort. The *p* value was calculated using the log-rank test. (**B**) Relapse-free survival according to median baseline lymphocyte-to-monocyte ratio in patients with histologic improvement and histologic activity. The *p* value was calculated using the log-rank test. (**C**) Relapse-free survival according to median baseline neutrophil-to-platelet ratio in the overall cohort. The *p* value was calculated using the log-rank test. (**D**) Relapse-free survival according to median baseline neutrophil-to-platelet ratio in patients with histologic improvement and activity. The *p* value was calculated using the log-rank test.

Kaplan–Meier curves demonstrated clinical relapse-free survival according to baseline NLR in all patients (Figure 2A). Relapse-free survival was significantly reduced in patients with a high baseline NLR in the histologically active subgroup (Figure 2B), whereas no significant difference was observed in the histologically improved subgroup. Kaplan–Meier analyses for LMR and NPR showed no significant differences in relapse-free survival, regardless of the histologic activity status (Figure 3).

## 4. Discussion

In this study, we investigated the prognostic significance of baseline NLR, LMR, and NPR for predicting relapse in patients with ulcerative colitis with MES 0 or 1, with a particular focus on the influence of histologic activity. Our key finding was that histologic activity markedly modified the prognostic value of the NLR. In patients with active histologic inflammation at baseline (the histologically active group), an elevated NLR was associated with a significantly higher risk of clinical relapse. In contrast, in patients with baseline histologic improvement, NLR was less discriminative of outcomes. Although endoscopic improvement may be observed if histological activity persists among these patients, an elevated NLR suggests a higher likelihood of relapse. Since our goal is to achieve histological healing, it is noteworthy that we can identify patients at a higher risk of relapse by assessing inflammatory markers, even among those who have not yet achieved it. To the best of our knowledge, this is the first study to demonstrate an interaction between histological disease activity and NLR, LMR, and NPR in predicting UC relapse in patients with MES scores of 0 or 1.

Our findings extend the growing literature on NLR as a prognostic marker for IBD. Prior studies have shown that the baseline NLR can predict response durability to therapy. For example, Nishida et al. reported that UC patients with a high NLR prior to infliximab treatment had a significantly higher chance of losing response, with a sensitivity of 78.6% and specificity of 78.3% for predicting relapse on anti-TNF therapy, and NLR remained an independent predictor in multivariate analysis of loss of response (LOR) (HR = 3.86) [10]. A high NLR has also been associated with poor long-term remission after calcineurin inhibitor therapy. Similarly, with regard to infliximab, one study reported that a pre-treatment NLR lower than 4.068 predicted a sustained response to a 52-week course of infliximab therapy in Crohn’s disease [10]. NLR may be a biomarker for predicting the outcome of systemic corticosteroid therapy [22]. These observations align with our finding that an elevated NLR indicates a worse prognosis, even outside the context of specific drug therapy. More recently, Kurimoto et al. focused on UC patients in endoscopic remission (MES 0–1) and demonstrated that those with NLR above 1.98 had significantly shorter relapse-free survival (45% relapsed over 46.4 months in the high NLR group vs. 31% in the low NLR group [7]). They calculated an adjusted hazard ratio of 1.74 (95% CI 1.02–2.98) for clinical relapse with a high NLR, which is consistent with our multivariable estimates. In our study, we went beyond simply analyzing MES and conducted a more specific analysis, including histological activity. Notably, our study adds that this prognostic signal of NLR is especially relevant when the colon appears to be histologically active. In patients who already have an active histology, the risk of relapse is elevated across the board, and a high NLR may simply reflect the same underlying inflammatory state that biopsies capture. In cases of histologic improvement, the prognostic role of NLR appears to be limited.

The LMR and NPR were evaluated as potential predictors. We observed that a low LMR (indicating higher monocyte counts relative to lymphocytes) in the histologically active subgroup tended to be associated with a higher relapse risk, although this relationship was not as strong as that of the NLR. This trend is biologically plausible; an elevated monocyte count has been linked to active disease in UC, and monocytes circulate as precursors of pro-inflammatory macrophages in the gut. Cherfane et al. previously noted that UC patients with monocytosis and a low LMR are more likely to have disease flares [14]. Our study suggests that a low LMR may similarly reflect the propensity for relapse, but larger samples are needed to confirm this as an independent prognostic marker. Neutrophil-to-platelet ratio (NPR) was not a significant predictor of relapse in our cohort. This index was introduced by Yamamoto-Furusho et al. as a novel method to gauge UC activity based on the hypothesis that platelets (acute-phase reactants) amplify neutrophil-driven inflammation [20]. In their cross-sectional study, the NPR was correlated with the clinical and endoscopic severity of UC. However, our longitudinal results imply that an elevated NPR at baseline does not independently predict relapse once other factors (including NLR and histology) are considered. It is possible that platelet counts in our patients were largely normal during improvement and rose in parallel with neutrophils during active disease, making NPR less distinctive than NLR alone. It is also plausible that the NPR could be more useful in specific contexts (e.g., acute severe colitis), which was not the focus of this study. Overall, the NLR appeared to be the most robust and practical of the three indices for prognostication in UC.

An important aspect of our study is the confirmation that histological inflammation remains a critical predictor of outcomes. Consistent with prior reports, we found that patients with active histological disease (even if endoscopically mild) had a substantially higher likelihood of relapse than those with histologic improvement [23]. This underscores the fact that histologic healing is associated with deeper remission and better long-term outcomes. For instance, one study showed that among patients with endoscopic remission, the relapse rate was fourfold lower if histologic normalization was present than without histologic normalization (50% vs. 12% over two years) [24]. Our data support this finding, as histologically active patients have worse relapse-free survival. A novel insight from our analysis is how histology and NLR can be integrated; patients with histologically active disease and an elevated NLR might have a higher relapse risk, particularly in those with mild residual histologic activity. In contrast, a patient with both histologic remission and a low NLR appears to have an extremely favorable prognosis. This interplay suggests that combining the mucosal healing assessment with a systemic inflammation marker could enhance risk stratification. This also raises a practical point: patients in endoscopic and histological remission are often considered for therapy de-escalation; however, those with biomarker evidence of inflammation (high NLR or other indices) should be monitored more closely or even continued on aggressive maintenance to preempt a flare.

Although high baseline NLR was associated with shorter relapse-free survival in patients with histologic activity, the early portion of the Kaplan–Meier curve showed some overlap between the low- and high-NLR groups, and early relapse events were also observed among patients with low NLR. This finding suggests that a low NLR does not completely exclude relapse risk, particularly when histologic activity persists. Histologic activity itself represents residual microscopic inflammation and may confer relapse risk even in patients with a favorable systemic inflammatory marker profile. Therefore, NLR should be interpreted as an adjunctive marker for risk stratification rather than as a stand-alone predictor of relapse.

The biological rationale for why a high NLR predicts relapse can be speculated. Neutrophils are front-line effector cells in UC’s inflammatory cascade that infiltrate the colonic mucosa, where they release proteases and reactive oxygen species, and form neutrophil extracellular traps (NETs) that can damage tissues [25,26]. An elevated neutrophil count (reflected by the NLR) likely indicates ongoing low-grade immune activation, even in the absence of clinical symptoms. Furthermore, relative lymphopenia (another component of NLR) might signal stress, corticosteroid use, or impaired adaptive immune regulation. Similarly, an increase in circulating monocytes (low LMR) can predispose patients to relapse by providing a reservoir of cells that can migrate to the gut and perpetuate inflammation. Platelet count, a potential biomarker, correlates with the degree of endoscopic inflammation and disease recurrence in UC patients and is significantly higher in patients with active UC than in those in the remission stage [27]. Although we did not find the NPR to be independently predictive, the role of platelets should not be dismissed; thrombocytosis in UC has been associated with disease severity and thromboembolic risk [8].

This study had some limitations. First, this was a single-center study, and although the sample size was moderate, the number of relapse events in the subgroups was relatively small. However, a standardized protocol for ulcerative colitis has been implemented and maintained over a prolonged period at our institution, enabling consistent approaches to patient diagnosis, treatment, and monitoring. This could affect the stability of the multivariable models and interaction analyses. Nevertheless, larger multicenter cohorts would strengthen confidence in the observed effect modification by histologic activity. Second, this retrospective cohort study introduced potential selection bias and residual confounding factors that could not be fully eliminated. Third, we focused on the baseline index values and did not incorporate dynamic changes over time. It is conceivable that trends in laboratory findings during follow-up (or at the time of endoscopic remission) might further enhance the predictive accuracy, as suggested in other chronic inflammatory conditions. Fourth, while we dichotomized histologic activity as active vs. inactive for simplicity, histologic disease can be graded on a spectrum. More granular scoring (e.g., using the Nancy or Robarts Histopathology Indices) might better quantify risk. Nevertheless, our dichotomy reflects the real-world clinical question of whether any residual inflammation is present, which is highly relevant to patient counseling and treatment decisions. Finally, we did not directly compare blood indices with fecal markers such as calprotectin. Fecal calprotectin is an established predictor of relapse in UC, including patients with mucosal healing. In practice, a combination of fecal and blood biomarkers may provide complementary information. For instance, a patient in clinical remission with normal calprotectin and a low NLR would be reassuring, whereas discrepancies (e.g., low calprotectin but high NLR) might prompt closer scrutiny.

Despite these limitations, our study provides clinically relevant insights. The NLR is an inexpensive and widely available test that can be incorporated into routine UC follow-up. Our data suggest that measuring the NLR at the time of endoscopic assessment could help identify patients at high risk of relapse. Patients with histologically inactive disease and a low NLR appear to maintain durable remission and might avoid overtreatment, whereas those with either active histology or an elevated NLR (or both) may benefit from optimized therapy and closer monitoring. For example, rather than tapering therapy in all endoscopically healed patients, a clinician might choose to continue full-dose treatment in a patient whose biopsies show no active colitis but whose NLR is 3 or 4, on the premise that subclinical inflammation may persist. In contrast, a patient with complete histological improvement and NLR < 2 might be a candidate for gradual de-escalation with careful observation. This approach aligns with the concept of personalized medicine, tailoring treatment intensity to an individual’s risk profile.

## 5. Conclusions

Histological activity significantly modified the prognostic value of baseline NLR in patients with UC. Our results indicated that an elevated NLR is a warning sign of impending relapse, particularly in patients with histological inflammation. These findings reinforce the importance of achieving deep remission (including at the histological level) and suggest that peripheral blood indices such as the NLR can serve as useful adjuncts for risk stratification. By integrating histological and hematological markers, clinicians may better personalize maintenance strategies, intensify therapy for those at high risk, and avoid unnecessary treatment in those at low risk. Ultimately, this approach could help to prolong remission and improve long-term outcomes in patients with colitis.

## Figures and Tables

**Table 1:** Baseline characteristics and clinical outcomes of patients according to histologic activity.

Variable	Total (*n* = 435)	Histologic Activity Geboes Score ≥ 3.1 (*n* = 132)	Histologic Improvement Geboes Score < 3.1 (*n* = 303)	*p* Value
Age, years	48 (37, 58)	43 (33, 56)	50 (38, 60)	<0.01
Male sex, *n* (%)	228 (52.4)	60 (45.5)	168 (55.4)	0.05
Age at diagnosis, years	39 (29–49)	36.5 (26.5, 46.5)	39 (30, 49)	
Disease duration, months	35.7 (25.00–45.00)	29.45 (23.25–39.90)	37.20 (26.45–45.75)	<0.05
Body mass index, kg/m^2^	22.8 (20.9, 25.0)	22.6 (20.6, 24.9)	22.9 (20.9, 25.0)	0.66
MES 1 at index colonoscopy, *n* (%)	245 (56.3)	118 (89.4)	127 (41.9)	<0.01
CRP, mg/dL	0.05 (0.03–0.11)	0.05 (0.03–0.11)	0.04 (0.03–0.11)	0.80
Current medication				
5-ASA, topical	86 (20.0)	35 (26.5)	51 (16.8)	0.02
5-ASA, oral	174 (40.0)	34 (25.8)	140 (46.2)	<0.01
5-ASA, both topical and oral	143 (33.0)	56 (42.4)	87 (28.7)	0.01
Steroid	6 (1.4)	2 (1.5)	4 (1.3)	1.00
Azathioprine	27 (6.2)	4 (3.0)	23 (7.6)	0.06
Immunomodulator	35 (8.1)	7 (5.3)	28 (9.3)	0.16
Baseline NLR	1.72 (1.23–2.22)	1.82 (1.35–2.19)	1.65 (1.21–2.24)	0.18
Baseline LMR	4.89 (3.71–6.15)	4.73 (3.70–6.20)	4.90 (3.72–6.07)	0.80
Baseline NPR	0.01 (0.01–0.02)	0.01 (0.01–0.02)	0.01 (0.01–0.02)	0.60
Clinical remission by PRO-2, n (%)	335 (77.0)	52 (39.4)	283 (93.4)	0.01

Data are presented as median (1st, 3rd quartile) or n (%). BMI, body mass index; MES, Mayo endoscopic score; NLR, neutrophil-to-lymphocyte ratio; LMR, lymphocyte-to-monocyte ratio; NPR, neutrophil-to-platelet ratio. *p*-values by Chi-squared test or Fisher’s exact test for categorical variables and Mann–Whitney test for continuous variables.

**Table 2:** Univariable Cox regression analyses for clinical relapse stratified by histologic activity.

Marker	Histologic Improvement Geboes Score < 3.1 (n = 303)			Histologic Activity Geboes Score ≥ 3.1 (n = 132)		
	HR	95% CI	*p* Value	HR	95% CI	*p* Value
NLR	0.98	0.697–1.375	0.901	1.72	1.062–2.921	0.028
LMR	1.105	0.786–1.553	0.566	0.818	0.508–1.315	0.406
NPR	0.982	0.699–1.379	0.916	1.293	0.804–2.077	0.289
Age	0.995	0.983–1.007	0.387	1.007	0.990–1.023	0.438
Age at diagnosis	0.991	0.978–1.003	0.147	1.010	0.993–1.027	0.258
Male sex	1.136	0.808–1.597	0.465	1.355	0.840–2.185	0.213
Disease duration, months	1.003	0.994–1.013	0.475	1.006	0.992–1.019	0.421
MES = 1	1.611	1.141–2.275	0.007	1.301	0.594–2.853	0.511
CRP	1.660	0.851–3.237	0.137	1.067	0.331–3.438	0.914

NLR neutrophil-to-lymphocyte ratio, LMR lymphocyte-to-monocyte ratio, NPR, neutrophil-to-platelet ratio, MES Mayo endoscopic score, CRP C-reactive protein.

**Table 3:** Multivariable Cox regression analyses for clinical relapse adjusted for Mayo Endoscopic Subscore.

Marker	Histologic Improvement Geboes Score < 3.1 (n = 303)			Histologic Activity Geboes Score ≥ 3.1 (n = 132)			*p* for Interaction
	HR	95% CI	*p* Value	HR	95% CI	*p* Value	
NLR	0.958	0.682–1.347	0.805	1.826	1.095–2.045	0.021	0.019
LMR	1.086	0.772–1.527	0.636	0.817	0.508–1.314	0.405	0.270
NPR	0.983	0.700–1.380	0.920	1.286	0.800–2.066	0.299	0.230

All models were adjusted for Mayo Endoscopic Subscore. NLR, neutrophil-to-lymphocyte ratio; LMR, lymphocyte-to-monocyte ratio; NPR, neutrophil-to-platelet ratio.

## Data Availability

The data underlying this article cannot be shared publicly due to the privacy of individuals who participated in the study. The data will be shared on reasonable request to the corresponding author.

## Abbreviations

5-ASA, 5-aminosalicylic acid; CI, confidence interval; CR, clinical remission; CRP, C-reactive protein; ESR, erythrocyte sedimentation rate; HR, hazard ratio; IBD, inflammatory bowel disease; IQR, interquartile range; LMR, lymphocyte-to-monocyte ratio; MES, Mayo Endoscopic Subscore; NLR, neutrophil-to-lymphocyte ratio; NPR, neutrophil-to-platelet ratio; PRO, patient-reported outcome; UC, ulcerative colitis; WBC, white blood cell.
